# Supplementary material for: Bacillus thuringiensis toxins divert progenitor cells toward enteroendocrine fate by decreasing cell adhesion with intestinal stem cells in Drosophila
Source: eLife. 2023 Feb 27;12:e80179. doi: 10.7554/eLife.80179 (PMC9977296; doi:10.7554/eLife.80179)
Supplement: Figure 6—source data 1. [file elife-80179-fig6-data1.zip › Figure 6 source data 1/Figure 6.pptx]

## Slide 1
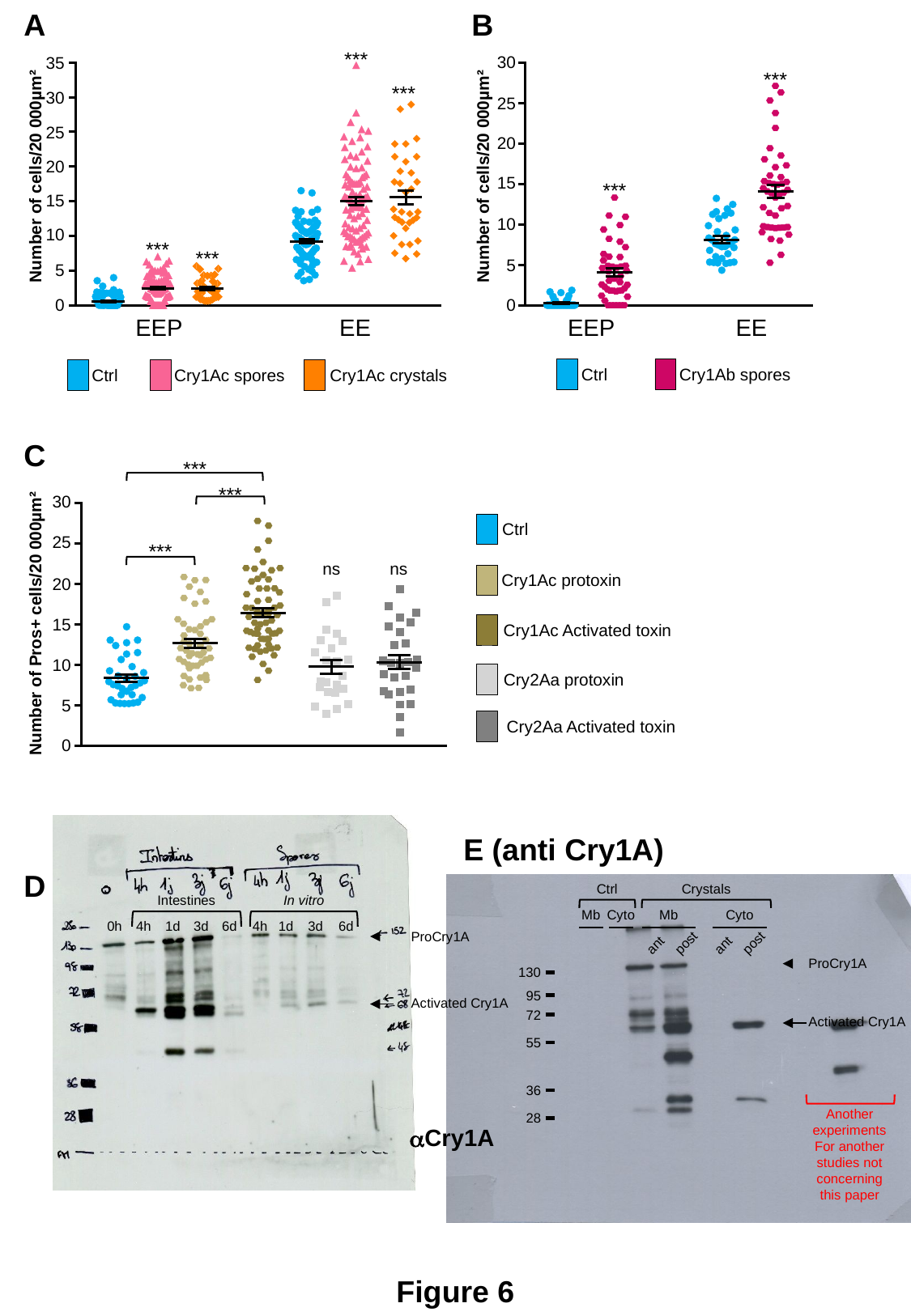

A
B
***
30
35
***
***
30
25
25
20
20
Number of cells/20 000µm²
Number of cells/20 000µm²
15
***
15
10
10
***
***
5
5
0
0
EEP
EE
EEP
EE
Ctrl
 Cry1Ab spores
Ctrl
 Cry1Ac spores
 Cry1Ac crystals
C
***
***
30
25
***
ns
ns
20
15
10
5
0
Ctrl
 Cry1Ac protoxin
Number of Pros+ cells/20 000µm²
 Cry1Ac Activated toxin
 Cry2Aa protoxin
 Cry2Aa Activated toxin
E (anti Cry1A)
D
Ctrl
Crystals
Intestines
In vitro
E (anti-Actin)
Mb
Cyto
Mb
Cyto
0h
4h
1d
3d
6d
4h
1d
3d
6d
ProCry1A
post
post
ant
ant
ProCry1A
130
95
72
55
36
28
Activated Cry1A
Activated Cry1A
Another experiments
For another studies not concerning this paper
aCry1A
n=24
Figure 6
