# Supplementary figures and images for: Bacillus thuringiensis toxins divert progenitor cells toward enteroendocrine fate by decreasing cell adhesion with intestinal stem cells in Drosophila
Source: eLife. 2023 Feb 27;12:e80179. doi: 10.7554/eLife.80179 (PMC9977296; doi:10.7554/eLife.80179)

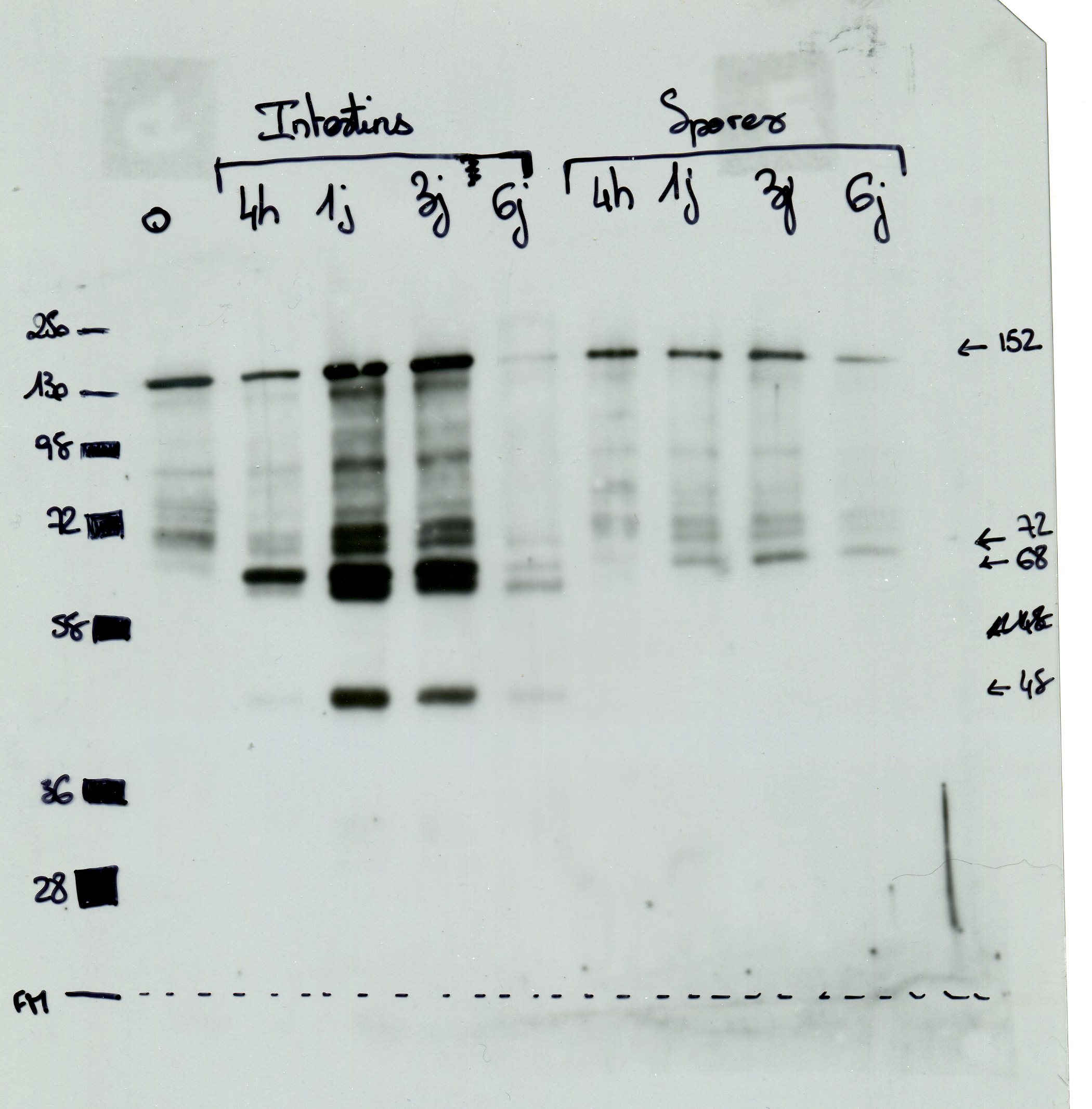

Supplement: Figure 6—source data 1. [file elife-80179-fig6-data1.zip › Figure 6 source data 1/6D.jpg]

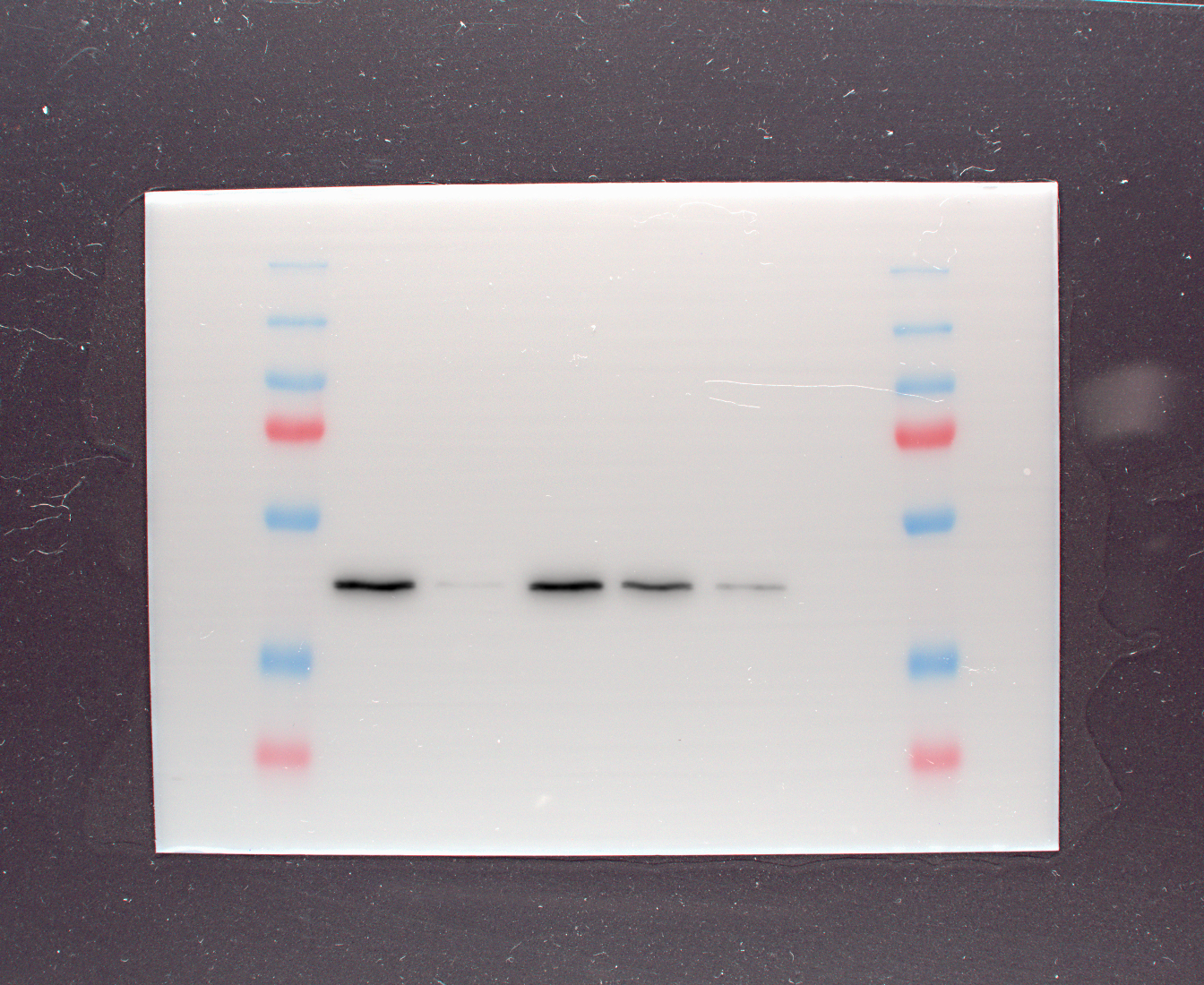

Supplement: Figure 6—source data 1. [file elife-80179-fig6-data1.zip › Figure 6 source data 1/6E actin1 chemi.Tif]

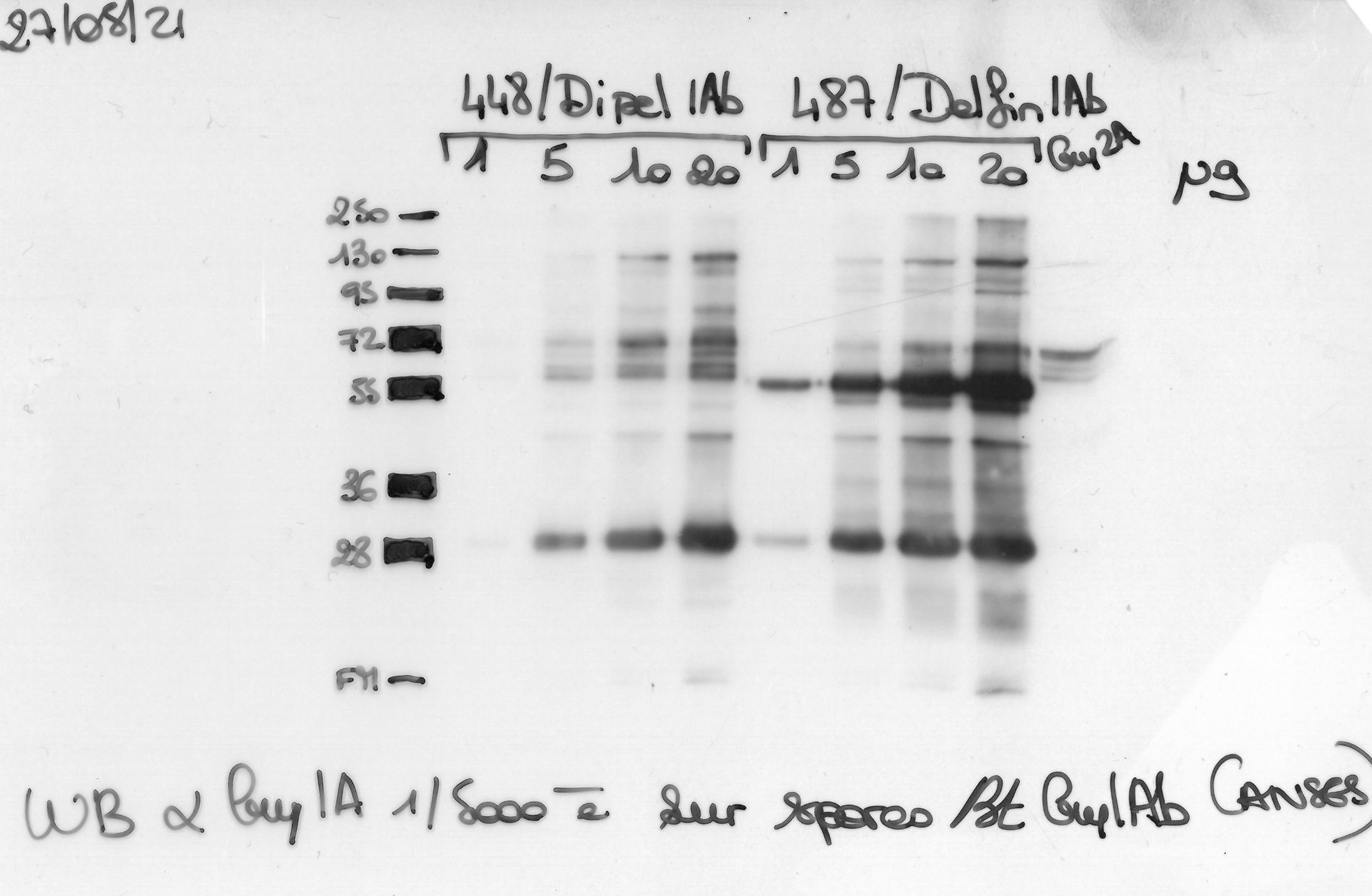

Supplement: Figure 6—figure supplement 1—source data 1. [file elife-80179-fig6-figsupp1-data1.zip › Figure 6 - fig sup 1 source data 1/Figure 6 - figure supplement 6I.tif]
