# Supplementary material for: Bacillus thuringiensis toxins divert progenitor cells toward enteroendocrine fate by decreasing cell adhesion with intestinal stem cells in Drosophila
Source: eLife. 2023 Feb 27;12:e80179. doi: 10.7554/eLife.80179 (PMC9977296; doi:10.7554/eLife.80179)
Supplement: Figure 6—figure supplement 1—source data 1. [file elife-80179-fig6-figsupp1-data1.zip › Figure 6 - fig sup 1 source data 1/Figure 6 - figure supplement 6 Data source 2.pptx]

## Slide 1
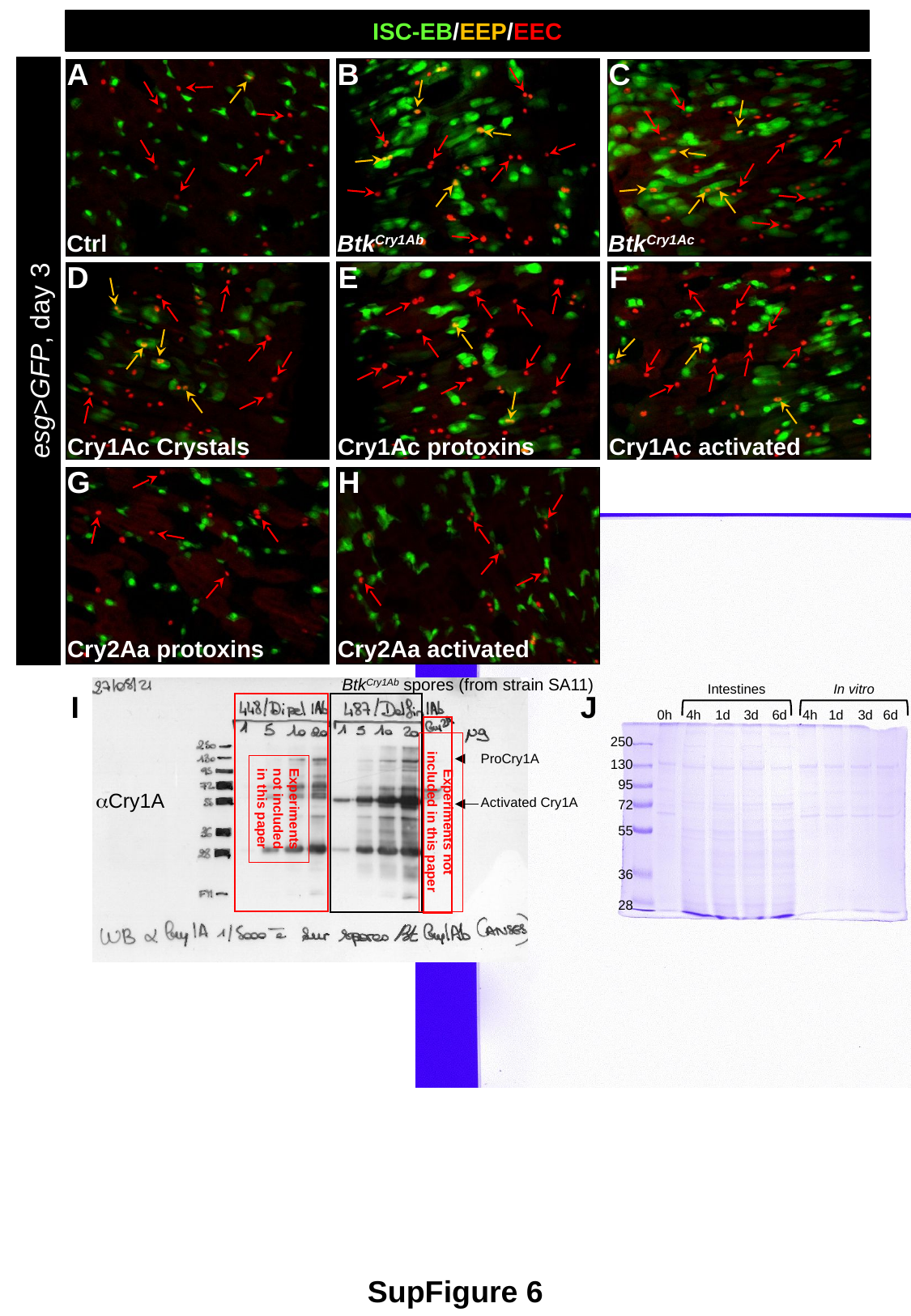

ISC-EB/EEP/EEC
A
B
C
Ctrl
BtkCry1Ab
BtkCry1Ac
D
E
F
esg>GFP, day 3
Cry1Ac Crystals
Cry1Ac protoxins
Cry1Ac activated
G
H
Cry2Aa protoxins
Cry2Aa activated
BtkCry1Ab spores (from strain SA11)
Intestines
In vitro
I
J
0h
4h
1d
3d
6d
4h
1d
3d
6d
250
ProCry1A
130
95
Experiments not included in this paper
aCry1A
Activated Cry1A
72
Experiments not included in this paper
55
36
28
SupFigure 6
